# Identification and Isolation of Active Compounds from *Astragalus membranaceus* that Improve Insulin Secretion by Regulating Pancreatic Β-Cell Metabolism

**DOI:** 10.3390/biom9100618

**Published:** 2019-10-17

**Authors:** Dahae Lee, Da Hye Lee, Sungyoul Choi, Jin Su Lee, Dae Sik Jang, Ki Sung Kang

**Affiliations:** 1 College of Korean Medicine, Gachon University, Seongnam 13120, Korea; pjsldh@naver.com (D.L.); pc1075@gachon.ac.kr (S.C.); 2 Department of Life and Nanopharmaceutical Sciences, Graduate School, Kyung Hee University, Seoul 02447, Korea; marylee93@naver.com (D.H.L.); lee2649318@naver.com (J.S.L.)

**Keywords:** *Astragalus membranaceus*, insulin, PI3K, AKT, PPARγ, PDX-1

## Abstract

In type 2 diabetes (T2D), insufficient secretion of insulin from the pancreatic β-cells contributes to high blood glucose levels, associated with metabolic dysregulation. Interest in natural products to complement or replace existing antidiabetic medications has increased. In this study, we examined the effect of *Astragalus membranaceus* extract (ASME) and its compounds **1**–**9** on glucose-stimulated insulin secretion (GSIS) from pancreatic β-cells. ASME and compounds **1**–**9** isolated from *A. membranaceus* stimulated insulin secretion in INS-1 cells without inducing cytotoxicity. A further experiment showed that compounds **2**, **3**, and **5** enhanced the phosphorylation of total insulin receptor substrate-2 (IRS-2), phosphatidylinositol 3-kinase (PI3K), and Akt, and activated pancreatic and duodenal homeobox-1 (PDX-1) and peroxisome proliferator-activated receptor-γ (PPAR-γ), which are associated with β-cell function and insulin secretion. The data suggest that two isoflavonoids (**2** and **3)** and a nucleoside (compound **5**), isolated from the roots of *A. membranaceus*, have the potential to improve insulin secretion in β-cells, representing the first step towards the development of potent antidiabetic drugs.

## 1. Introduction

Diabetes is characterized by high blood glucose levels (hyperglycemia) and is a common health problem that affects 387 million people worldwide [1]. It is an important risk factor for eye, kidney, nerve, and cardiovascular damage [2]. Type 2 diabetes (T2D) accounts for approximately 90% of all diabetes cases, in which insulin resistance is the primary pathogenic condition. It results in the failure of insulin action in metabolic target tissues, such as muscle, liver, and adipose tissues, due to the insufficient secretion of insulin from the pancreatic β-cells located within the islets of Langerhans [3,4]. A decline in the mass of the pancreatic β-cells, rate of insulin secretion from these cells, or a combination of both, leads to insufficient insulin secretion [5]. Thus, a study focused on preserving the secretory function and mass of the pancreatic β-cells is a strategic approach for the treatment of diabetes.

Interest in natural products to complement or replace existing antidiabetic medications has increased [6]. Approximately 50% of the drugs approved by the US Food and Drug Administration (FDA) are natural compounds or their derivatives, because currently available antidiabetic drugs, including insulin, metformin, and sulfonylureas, are often associated with adverse effects [7]. Some antidiabetic drugs, such as pycnogenol, a trademarked supplement for diabetes derived from the bark extract of the French maritime pine, *Pinus pinaster* Ait. [8], are obtained from natural products [9]. In addition, studies focusing on natural products and traditional medicines for new effective antidiabetic agents have been published [10,11]. Metformin leads to vitamin B12 or folate deficiency and anemia, especially in elderly patients [12,13]. Insulin and sulfonylureas are associated with hypoglycemia [6]. Although insulin lispro was developed in an effort to minimize the risk of hypoglycemia, the number of people with diabetes is predicted to increase to 592 million by 2035 [1]. This emphasizes the need to focus on agents that prevent and treat diabetes using natural products. *Astragalus membranaceus* extract and its constituents are derived from a plant known for its antidiabetic effect [14]. *A. membranaceus* is a Leguminosae flowering plant species used for diabetes treatment in Chinese herbal medicine [15]. It contains saponins, flavonoids, isoflavonoids, sterols, amino acids, polysaccharides, and volatile oils [14,15]. 

Recent research on T2D focuses on endogenous β-cell function, associated with the rate of insulin secretion from these cells [5]. In T2D models, saponins [16], flavonoids [17], and polysaccharides [18] regulate pancreatic β-cell proliferation and insulin-signaling pathways by enhancing the activation of peroxisome proliferator-activated receptor-γ (PPAR-γ), which is a transcription factor belonging to the nuclear receptor family and expressed in a variety of tissues [19]. The actions of PPAR-γ within pancreatic β-cells are directly involved in β-cell development and function, by regulating gene expression [20]. PPAR-γ directly regulates the nuclear translocation of pancreatic and duodenal homeobox 1 (PDX-1) [21]. PDX-1, a key transcription factor, then binds to the insulin promoter and regulates glucose-stimulated insulin transcription [22]. Additionally, insulin receptor substrate-2 (IRS-2) has important implications for the regulation of the maintenance of β-cell mass and function [23]. Increased expression of IRS-2 stimulates the phosphatidylinositol 3-kinase/protein kinase B (PI3K/Akt) pathway in pancreatic β-cells and promotes β-cell growth and survival. 

Here, we report for the first time, to our knowledge, the potential to improve insulin secretion in the INS-1 rat insulin-secreting β-cell line using isoflavonoids isolated from *A. membranaceus*, evaluated as a treatment strategy for T2D. Nine compounds (calycosin, calycosin-7-*O*-β-d-glucoside, formononetin, formononetin-7-*O*-β-d-glucoside, adenosine, 3-(β-d-ribofuranosyl)-2,3-dihydro-6H-1,3-oxazine-2,6-dione, acetylastragaloside I, astragaloside I, and astragaloside II) were isolated from the roots of *A. membranaceus* and identified. Additionally, the activities that improved glucose-stimulated insulin secretion (GSIS) and the underlying mechanisms of action were identified. This study demonstrated that two isoflavonoids and a nucleoside isolated from *A. membranaceus* result in the overexpression of IRS-2, PI3K, Akt, PDX-1, and PPAR-γ, which are associated with β-cell function and enhanced insulin secretion. 

## 2. Materials and Methods 

### 2.1. General Experimental Procedures

Column chromatography was performed on silica gel (70–230 and 230–400 mesh ASTM, Merck, Kenilworth, NJ, USA) and Diaion HP-20 (Mitsubishi Chemical Co., Tokyo, Japan). Flash chromatography was performed using the flash purification system (Combi Flash Rf, Teledyne Isco, Lincoln, NE, USA) with Redi Sep-C18 (26 g, 43 g Teledyne Isco). High-performance liquid chromatography (HPLC) was performed using the Gilson purification system, with a J’sphere ODS column (250 × 20.0 mm i.d., 4.0 μm, YMC Co., Tokyo, Japan). Thin-layer chromatography (TLC) analysis was performed on silica gel 60 F_254_ and RP-18 F_254S_ plates (Merck). Nuclear magnetic resonance (NMR) spectra were obtained using a JEOL 500 MHz, using tetramethylsilane as an internal standard, and chemical shifts were expressed as δ values. Organic solvents for the chromatographic separations and extractions were distilled before use.

### 2.2. Plant Material

The roots of *A. membranaceus* (Leguminosae) were purchased from Hyunjin Pharmaceutical Co. (Seoul, Korea), in January 2015. The origin of the plant material was identified by one of authors D.S.J., and a representative specimen (ASME1-2015) was deposited at the Laboratory of Natural Product Medicine, College of Pharmacy, Kyung Hee University, Seoul, Korea.

### 2.3. Extraction and Isolation

The dried roots of *A. membranaceus* (6.0 kg) were refluxed once with 60 L of 70% aqueous ethanol (EtOH) for 3 h, and the solvent was evaporated in vacuo at 45 °C to give a 70% EtOH extract (ASME). ASME (1.7 kg) was separated using Diaion HP-20 as the stationary phase and eluted with a MeOH-H_2_O gradient (from 0:1 to 1:0 *v*/*v*), ethyl acetate (EtOAc), and *n*-hexane, to produce seven fractions (K1–7). Fraction K2 (16.4 g) was fractionated using column chromatography (CC) over silica gel (70–230 mesh) and eluted with a gradient of CH_2_Cl_2_-MeOH-H_2_O (from 8:1.8:0.2 to 5:4.5:0.5 *v*/*v*) to produce five fractions (K2-1–5). Fraction K2-2 (2.6 g) was further separated into nine fractions (K2-2-1–9), using silica gel CC (230–400 mesh) with a stepwise gradient of CH_2_Cl_2_-MeOH-H_2_O (from 9:0.9:0.1 to 5:4.5:0.5 *v*/*v*). Adenosine (**5**, 20.7 mg) and 3-(β-d-ribofuranosyl)-2,3-dihydro-6H-1,3-oxazine-2,6-dione (**6**, 11.7 mg) were obtained from fraction K2-2-6 (240.0 mg) using a reversed-phase HPLC system with a J’sphere ODS column. Fraction K4 (13.0 g) was separated by silica gel CC (70–230 mesh) with a gradient of CH_2_Cl_2_-MeOH-H_2_O (from 8:1.8:0.2 to 5:4.5:0.5 *v*/*v*) to yield nine sub-fractions (K4-1–9). Calycosin-7-*O*-β-d-glucoside (**2**, 277.5 mg) was obtained by recrystallization of fraction K4-4 (1.5 g). Fraction K6 (22.3 g) was fractionated using silica gel CC (70–230 mesh) and eluted with a gradient of CH_2_Cl_2_-MeOH-H_2_O (from 8:1.8:0.2 to 7:2.7:0.3 *v*/*v*) to produce 14 fractions (K6-1–14). Formononetin (**3**, 5.1 g) was obtained from fraction K6-2. Formononetin-7-*O*-β-d-glucoside (**4**, 36.5 mg) and acetylastragaloside I (**7**, 25.5 mg) were obtained from fraction K6-8 (365.8 mg), with a flash chromatography system using a Redi Sep-C18 cartridge (26 g, MeOH-H_2_O, gradient from 0:10 to 9:1 *v*/*v*). Calycosin (**1**, 35.9 mg), astragaloside I (**8**, 249.1 mg), and astragaloside II (**9**, 71.9 mg) were isolated by repeated flash chromatography, using a Redi Sep-C18 cartridge (43 g, MeOH-H_2_O, 0:10 to 9:1 *v*/*v*, gradient), from fractions K6-3 (852. 8 mg), K6-9 (566.8 mg), and K6-12 (536.6 mg), respectively. 

### 2.4. Cell Culture

INS-1 cells (Biohermes, Shanghai, China), a rat insulin-secreting β-cell line, were cultured in a humidified atmosphere at 37 °C containing 5% CO_2_, in an RPMI-1640 medium (Cellgro, Manassas, VA, USA), supplemented with 2 mM L-glutamine, 0.05 mM 2-mercaptoethanol, 11 mM D-glucose, 1% penicillin/streptomycin (Invitrogen Co., Grand Island, NY, USA), 10% fetal bovine serum (FBS), 10 mM HEPES, and 1 mM sodium pyruvate.

### 2.5. Cell Viability 

An Ez-Cytox cell viability assay kit (Daeil Lab Service Co., Seoul, Korea) was used to measure cell viability [24]. To determine non-toxic dose ranges of ASME and compounds **1**–**9** isolated from *A. membranaceus*, INS-1 cells were cultured in 96-well plates for 24 h and, subsequently, treated with ASME and compounds **1**–**9** for 24 h. Ez-Cytox reagent (10 μL) was added and incubated for 2 h. Absorbance values at 450 nm were measured using a microplate reader (PowerWave XS, Bio-Tek Instruments, Winooski, VT, USA). 

### 2.6. GSIS Assay

An insulin secretion assay was used to determine the effect of ASME and compounds **1**–**9** on GSIS in INS-1 cells, using gliclazide as the positive control. To assess GSIS after treatment with ASME and compounds **1**–**9**, INS-1 cells were cultured in 12-well plates for 24 h, washed twice with Krebs–Ringer bicarbonate HEPES buffer and 2.8 mM glucose, and starved in fresh KRBB. After starvation for 2 h, the cells were treated with ASME, compounds **1**–**9**, and gliclazide. After 2 h, glucose (2.8 and 16.7 mM as basal and stimulant, respectively) was added to each well and incubated for 1 h. GSIS was measured using a rat insulin ELISA kit, as reported previously.

### 2.7. Western Blot Analysis

INS-1 cells were cultured in 6-well plates for 24 h and treated with compounds **2**, **3**, and **5** for 24 h to assess the levels of protein expression of PPAR-γ, PI3K, Akt, P-IRS-2 (Ser731), IRS-2, P-PI3K, P-Akt (Ser473), and PDX-1 after treatment. Western blot analysis was carried out to evaluate the expression of proteins related to pancreatic β-cell metabolism, as reported previously [25].

### 2.8. Statistical Analysis

Statistical significance was assessed using one-way analysis of variance (ANOVA) and multiple comparisons with a Bonferroni correction. *P* values less than 0.05 were considered statistically significant. All analyses were determined using SPSS Statistics ver. 19.0 (SPSS Inc., Chicago, IL, USA).

## 3. Results

### 3.1. Identification of Compounds **1**–**9**

**Figure 1:**
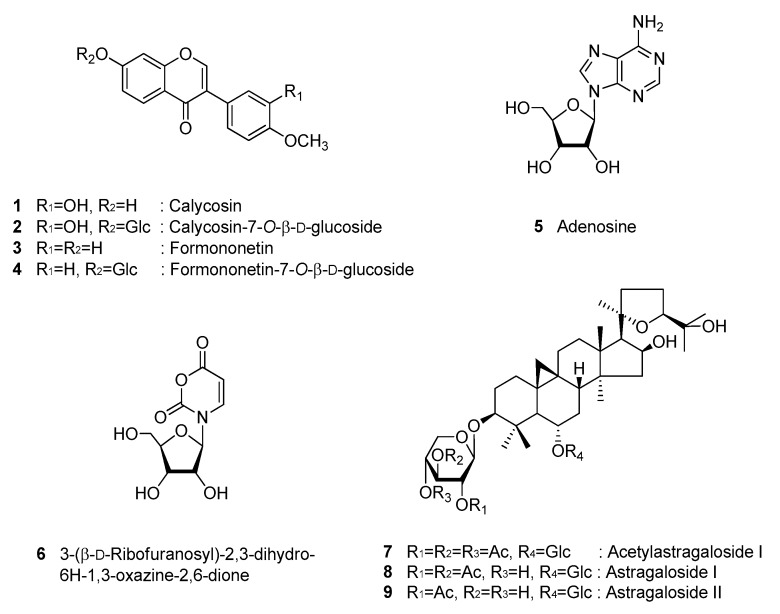
Chemical structures of compounds **1**–**9**.

Nine compounds (**1**–**9**) were isolated from the roots of *A. membranaceus* in this study. The purity of the isolates (>95%) was determined by NMR. The chemical structures of compounds **1**–**9** were identified as calycosin (**1**) [26], calycosin-7-*O*-β-d-glucoside (**2**) [27], formononetin (**3**) [28], formononetin-7-*O*-β-d-glucoside (**4**) [29], adenosine (**5**) [30], 3-(β-d-ribofuranosyl)-2,3-dihydro-6H-1,3-oxazine-2,6-dione (**6**) [31], acetylastragaloside I (**7**) [32], astragaloside I (**8**) [32], and astragaloside II (**9**) [33], by 1D and 2D NMR spectroscopic data and by comparison with published values (Figure 1). 

### 3.2. Effect of ASME and Compounds **1**–**9** on GSIS

**Figure 2:**
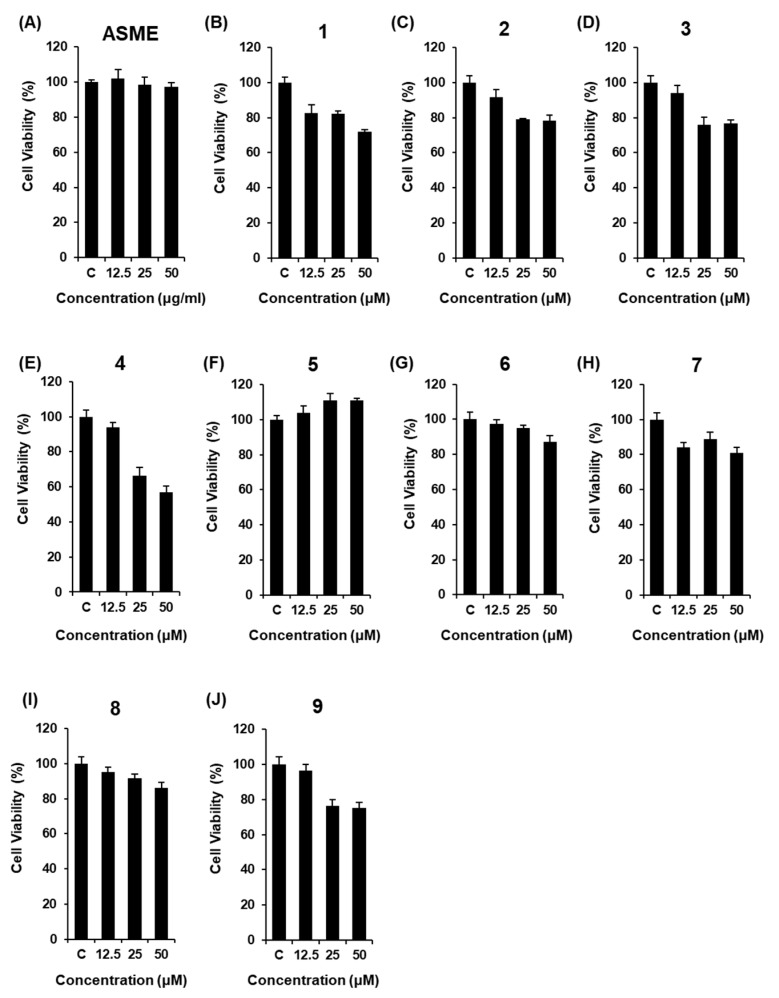
Effect of *A. membranaceus* extract (ASME) and compounds **1**–**9** isolated from *A. membranaceus*, on the viability of INS-1 cells. Effect of (**A**) ASME and (**B**–**J**) compounds **1**–**9**, compared with the control (0 μg/mL), on the viability of INS-1 cells for 24 h by MTT assay.

**Figure 3:**
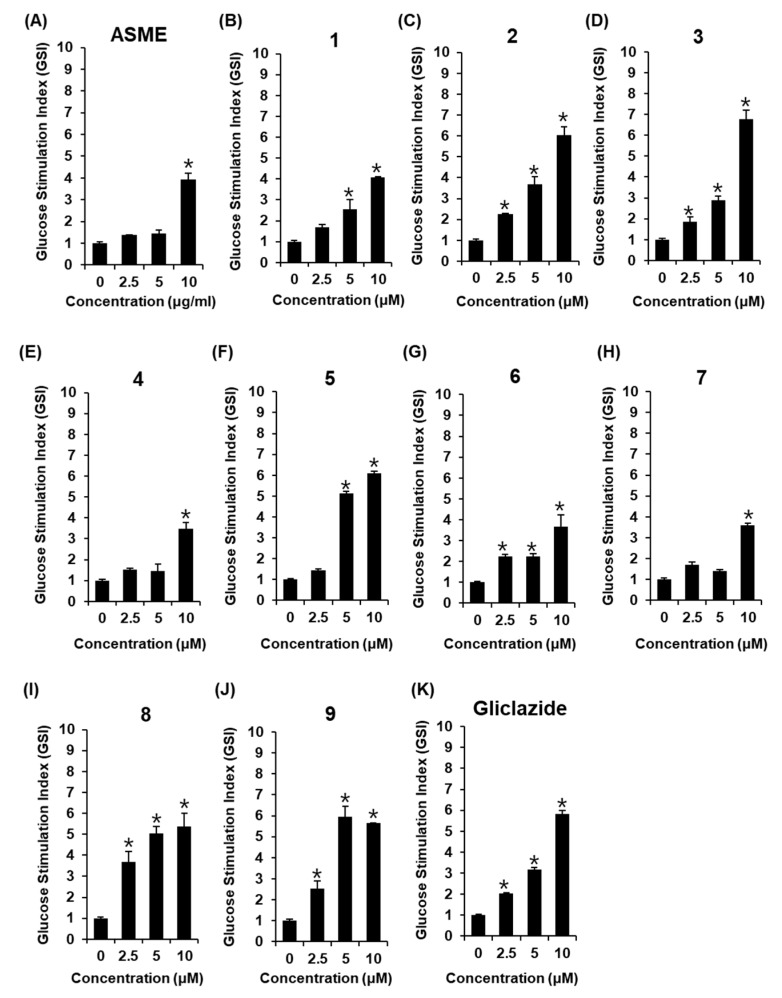
Effect of *A. membranaceus* extract (ASME) and compounds **1**–**9** isolated from *A. membranaceus* on glucose-stimulated insulin secretion (GSIS) in INS-1 cells. Effect of (**A**) ASME, (**B**–**J**) compounds **1**–**9**, and (**K**) gliclazide (positive control) on GSIS in INS-1 cells for 1 h by insulin secretion assay. * *p* < 0.05 compared to the control (0 μM).

The non-toxic dose of ASME and compounds **1**–**9** was determined using a cell viability assay on INS-1 cells. Although ASME did not show any toxic effect at 12.5, 25, and 50 μM (Figure 2A), some compounds isolated from ASME were slightly cytotoxic at concentrations of 25 μM and above, as cell viability decreased to below 80% (Figure 2B–E,J). ASME at 2.5, 5, and 10 μg/mL, and compounds **1**–**9** at 2.5, 5, and 10 μM, used in the insulin secretion assay, were based on the results of the cell viability assay. As shown in Figure 3A, ASME led to an increase in GSI in a dose-dependent manner. The GSI levels were 1.37 ± 0.01, 1.43 ± 0.17, and 3.92 ± 0.31 for ASME at 2.5 μg/mL, 5 μg/mL, and 10 μg/mL, respectively. Among compounds isolated from ASME, two isoflavonoids calycosin-7-*O*-β-d-glucoside (**2**), formononetin (**3**), and nucleoside adenosine (**5**) led to a significant increase in GSI in a dose-dependent manner (Figure 3C,D,F). The GSI levels were 6.03 ± 0.41, 6.77 ± 0.43, and 5.97 ± 0.10 for compounds **2**, **3**, and **5** at 10 μM, respectively. These GSI levels were similar to the GSI level of gliclazide at the same concentration (Figure 3K). The GSI levels were 2.03 ± 0.03, 3.16 ± 0.10, and 5.81 ± 0.16 for gliclazide at 2.5 μM, 5 μM, and 10 μM, respectively. Compounds **2**, **3**, and **5** stimulated insulin secretion in INS-1 cells without inducing cytotoxicity.

### 3.3. Effect of Compounds **2**, **3**, and **5** Isolated from A. membranaceus on the Protein Expression of PPARγ, P-IRS-2, IRS-2 (Ser731), P-PI3K, PI3K, P-Akt (Ser473), Akt, and PDX-1

**Figure 4:**
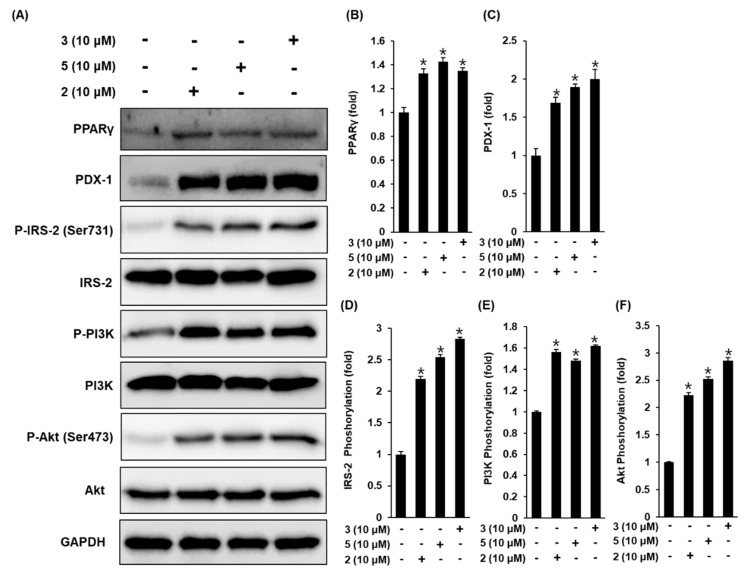
Effect of compounds **2**, **3**, and **5** isolated from *A. membranaceus* on the protein expression levels of peroxisome proliferator-activated receptor-γ (PPAR-γ), pancreatic and duodenal homeobox-1 (PDX-1), P-insulin receptor substrate-2 (IRS-2) (Ser731), IRS-2, P-phosphatidylinositol 3-kinase (PI3K), PI3K, P-Akt (Ser473), and Akt in INS-1 cells. (**A**) Protein expression levels of PPAR-γ, PDX-1, P-IRS-2 (Ser731), IRS-2, P-PI3K, PI3K, P-Akt (Ser473), Akt, and glyceraldehyde 3-phosphate dehydrogenase (GAPDH) in INS-1 cells treated or untreated with 10 μM compounds **2**, **3**, and **5** for 24 h. (**B**–**F**) Each bar graph presents the densitometric quantification of Western blot bands. * *p* < 0.05 compared to the control (0 μM).

**Figure 5:**
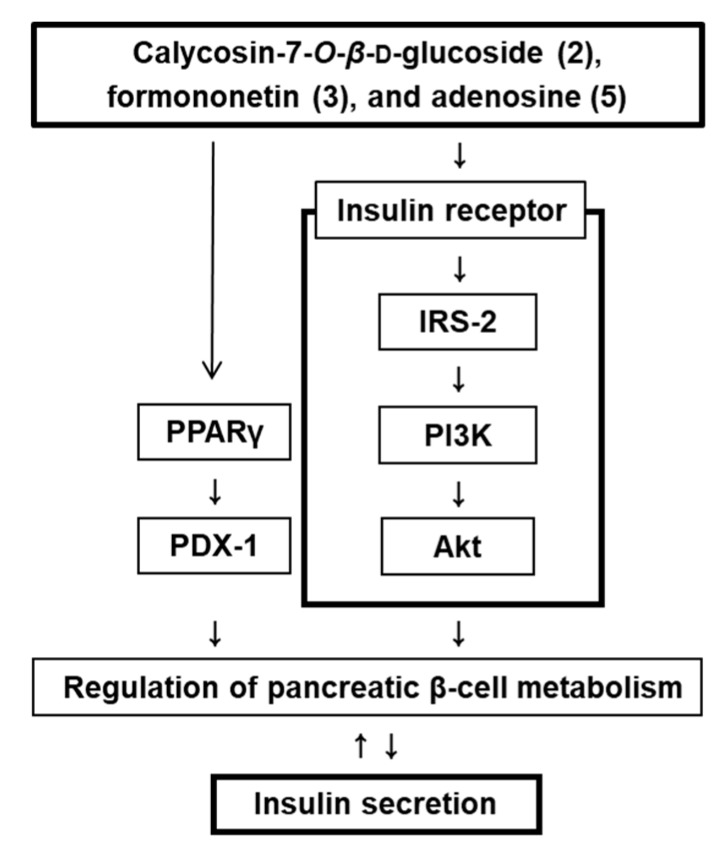
Schematic illustration of the effects of compounds **2**, **3**, and **5** isolated from *A. membranaceus* on the protein expression levels of peroxisome proliferator-activated receptor-γ (PPAR-γ), pancreatic and duodenal homeobox-1 (PDX-1), insulin receptor substrate-2 (IRS-2), phosphatidylinositol 3-kinase (PI3K), P-Akt (Ser473), and Akt in INS-1 cells.

To show the role of PPAR-γ, IRS-2, PI3K, Akt, and PDX-1 in the effect of compounds **2**, **3**, and **5** on GSIS, we measured these protein levels in pancreatic β-cell metabolism, and demonstrated that the protein expression levels of PPAR-γ, P-IRS-2 (Ser731), P-PI3K, P-Akt (Ser473), and PDX-1 increase upon treatment with compounds **2**, **3**, and **5** at 10 μM, compared to untreated controls (Figure 4). A schematic illustration of the proposed mechanism of the effects of compounds **2**, **3**, and **5** in pancreatic β-cell metabolism was shown in Figure 5.

## 4. Discussion

This study demonstrated that ASME and the compounds identified in this extract exerted insulin secretory effects. ASME and some active compounds isolated from ASME have been reported for their antidiabetic properties [14]. In an STZ-induced rat model of T1D, after treatment with astragalus polysaccharides [34,35,36] and soy isoflavones [36] isolated from ASME, glucose homeostasis was improved, but not by enhancing the capacity of insulin secretion levels [34,35]. In our study, ASME enhanced GSIS without inducing cytotoxicity in INS-1 cells. From this extract, nine compounds (**1**–**9**) including calycosin, calycosin-7-*O*-β-d-glucoside, formononetin, formononetin-7-*O*-β-d-glucoside, adenosine, 3-(β-d-ribofuranosyl)-2,3-dihydro-6H-1,3-oxazine-2,6-dione, acetylastragaloside I, astragaloside I, and astragaloside II, were isolated and identified. Among the isolated compounds, calycosin-7-*O*-β-d-glucoside (**2**), formononetin (**3**), and adenosine (**5**) led to a significant increase in GSI in a dose-dependent manner, without inducing cytotoxicity in INS-1 cells. The effects of these three compounds were similar to the effect of gliclazide, a medicine used to treat T2D and classified in the sulfonylurea class of insulin secretagogues [37], which improved pancreatic β-cell sensitivity to glucose and enhanced insulin secretion in clinical studies [38]. These results suggest that sufficient secretion of insulin after treatment with ASME and its bioactive compounds, in response to an increase of glucose, may inhibit characteristic diabetic hyperglycemia and improve the sensitivity of pancreatic β-cells to glucose. 

The most effective isoflavonoid identified in our study, formononetin (**3**), has been reported for its antidiabetic effect. In an experimental model of T2D, treatment with formononetin improves insulin sensitivity and reduces hyperglycemia by activating sirtuin 1 (SIRT1), an important regulator of energy metabolism that is involved in the regulation of insulin production and sensitivity and controls co-regulators, such as nuclear factor-kappa B (NF-κB), FOXO proteins, and PPAR-γ in pancreatic β cells [39]. In our previous study, the mechanisms of action mediating insulin secretion by compounds **2**, **3**, and **5** were evaluated. PPAR-γ is an important regulator of glucose metabolism by regulating gene expression [40], and is activated by compounds **2**, **3**, and **5**. Thiazolidinediones (TZDs), PPARγ agonists, are widely used antidiabetic drugs, but have side effects, including weight gain and hepatic dysfunction. Because of these side effects, a variety of natural compounds, including stilbenes, flavonoids, neolignans, sesquiterpenes, amorfrutins, and coumarins have been identified as PPAR-γ agonists in an attempt to increase the effectiveness of PPAR-γ, while limiting its side effects [41]. 

PPAR-γ binds to the PDX-1 promoter to upregulate PDX-1 expression, which is associated with pancreatic development and the capacity of β-cells [42,43]. In human pancreatic β-cells, PDX-1 mRNA levels are increased in the presence of gliclazide [44], which has a similar insulin secretory capacity to compounds **2**, **3**, and **5**. These three active compounds also increase the protein expression of PDX-1. Pancreatic β-cell malfunction is characterized by the lack of insulin production and secretion to regulate glucose metabolism, which results in hyperglycemia in PDX-1 knockout mice [45] and IRS-2 knockout mice [46]. IRS-2 is a member of a family of large adaptor proteins, linking insulin receptors to the activation of the PI3K/Akt pathway, which plays an important role in β-cell function [47,48]. Upregulation of IRS-2, PI3K, and Akt leads to the proliferation of these proteins in pancreatic β-cells, maintaining functional β-cell mass and enhancing insulin secretion [49]. In pancreatic islets isolated from patients with T2D, these expression levels are reduced [50,51]. We investigated the role of IRS-2 in the presence of compounds **2**, **3**, and **5**. IRS-2 phosphorylation at Ser731 was increased by compounds **2**, **3**, and **5**. In addition, PI3K-dependent Akt phosphorylation at Ser473 was observed after treatment with compounds **2**, **3**, and **5**. 

Consequently, the three active compounds (**2**, **3**, and **5**) facilitated insulin secretion by enhancing the expression of IRS-2, PI3K, Akt, PPAR-γ, and PDX-1. Despite these findings, further investigation is needed to determine how inhibitors of IRS-2, PI3K, Akt, PPAR-γ, and PDX-1 affect GSIS. Studies on the solubility, membrane permeability, absorption, distribution, and metabolism of the active compounds in vivo are also required, because these factors limit the oral bioavailability of the compounds [52,53]. In addition, in vivo studies of T2D are required to assess the antidiabetic potential of the active compounds, because their effects on insulin secretion from the pancreatic β cells may not be as significant upon oral administration. Clarifying the underlying mechanisms of action through further investigation may lead to the development of new drugs to prevent or delay the development of diabetes in patients who do not adequately respond to currently available antidiabetic drugs.

## 5. Conclusions

Thus, calycosin-7-*O*-β-d-glucoside (**2**), formononetin (**3**), and adenosine (**5**), isolated from the roots of *A. membranaceus*, were observed to potentiate GSIS from the pancreatic β-cells. Moreover, our results suggested that IRS-2, PI3K, Akt, PPAR-γ, and PDX-1 played important roles in these effects, highlighting the potential of these 3 active compounds in antidiabetic research.
